# Acute hypoxaemic respiratory failure after treatment with lower tidal volume ventilation facilitated by extracorporeal carbon dioxide removal: long-term outcomes from the REST randomised trial

**DOI:** 10.1136/thorax-2022-218874

**Published:** 2022-10-05

**Authors:** Andrew J Boyle, Clíona McDowell, Ashley Agus, Danielle Logan, Jonathan D Stewart, Colette Jackson, Jeanette Mills, James J McNamee, Daniel F McAuley

**Affiliations:** 1 Wellcome-Wolfson Institute for Experimental Medicine, Queen's University Belfast, Belfast, UK; 2 Regional Intensive Care Unit, Royal Victoria Hospital, Belfast, UK; 3 Northern Ireland Clinical Trials Unit, Belfast, UK

**Keywords:** ARDS

## Abstract

**Introduction:**

Lower tidal volume ventilation, facilitated by veno-venous extracorporeal carbon dioxide removal (vv-ECCO_2_R), does not improve 90-day mortality in patients with acute hypoxaemic respiratory failure (AHRF). The aim of this analysis was to evaluate the effect of this therapeutic strategy on long-term outcomes.

**Methods:**

This was a prespecified analysis of the REST trial, a UK-wide multicentre randomised clinical trial that compared lower tidal volume ventilation, facilitated by vv-ECCO_2_R (intervention), with standard care in the treatment of patients with moderate-to-severe AHRF. Mortality to 2 years was assessed, while respiratory function, post-traumatic stress disorder, cognitive function and health-related quality of life were evaluated in survivors at 1 year using standardised questionnaires.

**Results:**

Of 412 patients enrolled into the REST trial, 391 (95%) had 2-year mortality outcome data available. There was no difference in the time to death between intervention and standard care (HR 1.08 (0.81, 1.44); log-rank test p=0.61). 161 patients alive at 1 year provided at least one questionnaire response. There was no difference in respiratory function, post-traumatic stress disorder, cognitive dysfunction or health-related quality of life between patients allocated to intervention or standard care.

**Conclusion:**

Lower-tidal volume ventilation facilitated by vv-ECCO_2_R does not affect 1-year mortality in patients with moderate-to-severe AHRF. Of the patients who provided questionnaire responses, there was no treatment effect on long-term respiratory function, post-traumatic stress disorder, cognitive dysfunction or health-related quality of life.

**Trial registration number:**

ClinicalTrials.gov identifier: NCT02654327.

**WHAT IS ALREADY KNOWN ON THIS TOPIC**

- Patients admitted to the intensive care unit with acute hypoxaemic respiratory failure (AHRF) have significant long-term morbidity and mortality. The use of veno-venous extracorporeal carbon dioxide removal (vv-ECCO_2_R) to facilitate lower tidal volume ventilation does not improve 90-day mortality, but the effects on long-term mortality and functional outcomes are unclear.

**WHAT THIS STUDY ADDS**

- In this prespecified analysis of the REST trial, lower-tidal volume ventilation, facilitated by vv-ECCO_2_R, does not affect long-term mortality, or other long-term outcomes in patients with moderate-to-severe AHRF who completed follow-up.

**HOW THIS STUDY MIGHT AFFECT RESEARCH, PRACTICE OR POLICY**

- These findings reinforce that a lower tidal volume ventilation strategy, facilitated by vv-ECCO_2_R as delivered in the REST trial, should not be used in the management of patients with moderate-to-severe AHRF, and that vv-ECCO_2_R should not be used routinely outside the setting of clinical trials.

## Introduction

Acute hypoxaemic respiratory failure (AHRF) is one of the most frequent indications for admission to the intensive care unit (ICU), and is associated with hospital inpatient mortality rates of approximately 40%.^1^ In addition to this high short-term mortality, patients with AHRF are also at increased risk of death in the 2-year period following hospital admission.^2^

Acute respiratory distress syndrome (ARDS) is a common cause of AHRF, and survivors of ARDS have a significant and persistent functional limitation up to 5 years after discharge from the ICU, with over 50% of patients not returning to work.^3–6^ For survivors of critical care admission, the prevalence of psychiatric symptoms, including PTSD and anxiety, may be greater than 60%, while cognitive impairment affects up to 60% of patients with ARDS who survive to 1 year.^7 8^ Mechanical ventilation can induce hippocampal neuronal cell apoptosis,^9^ and preclinical models suggest that this may be exacerbated by higher tidal volume ventilation.^10^ It is therefore feasible that strategies aimed at reducing the tidal volume delivered as part of mechanical ventilation for patients with AHRF may reduce the long-term psychological and cognitive impact in patients with AHRF who survive hospital admission.

The short-term and long-term outcomes from AHRF emphasise the importance of continuing to identify novel therapies that may improve these. The protective ventilation with veno-venous lung assist in respiratory failure (REST) trial evaluated the use of veno-venous extracorporeal carbon dioxide removal (vv-ECCO_2_R) (using a device with maximal blood flow rate of up to 550 mL/min and CO_2_ removal capacity of approximately 80–90 mL/min^11^) to facilitate lower tidal volume ventilation in patients with AHRF.^12^ The REST trial aimed to achieve mechanical ventilation with a tidal volume of ≤3 mL/kg predicted body weight (PBW), while the comparator group received standard care. The REST trial was discontinued by the data monitoring and ethics committee prior to recruitment of the planned sample size, and the results of the trial did not demonstrate any short-term benefit of lower tidal volume ventilation facilitated by vv-ECCO_2_R. Given that AHRF is associated with increased risk of death for up to 2 years,^2^ and adherence to a lung-protective ventilation strategy is associated with improved long-term outcomes in ARDS,^13^ it is feasible that analysis of the long-term outcomes of patients recruited to the REST trial may identify benefit from the intervention strategy. Furthermore, it is uncertain whether lower tidal volume ventilation, below current standards of care,^14 15^ is associated with long-term improvements in functional status of survivors of AHRF. The aim of this analysis was to assess whether lower tidal volume ventilation, facilitated by vv-ECCO_2_R, was associated with a reduction in long-term mortality, and whether it improved long-term respiratory, psychological and cognitive function, or health-related quality of life, in survivors of AHRF.

## Methods

### Study design

This was a planned secondary analysis of the REST trial.^16^ The REST trial was a multicentre, randomised, allocation concealed, open, pragmatic clinical trial, conducted across 51 ICUs in the UK, evaluating lower tidal volume ventilation facilitated by ECCO_2_R with standard care. Between May 2016 and December 2019, 412 participants were enrolled. Patients were eligible for the REST trial if they were within 48 hours of onset of an acute and potentially reversible cause of hypoxaemic respiratory failure (defined as a ratio of the partial pressure of oxygen in arterial blood to the fractional inspired concentration of oxygen (PF) ratio<150 mm Hg) while receiving invasive mechanical ventilation with a positive end-expiratory pressure (PEEP) of at least 5 cm H_2_O.

In patients randomised to receive intervention, vv-ECCO_2_R was commenced via a percutaneous catheter inserted into a central vein. Intravenous heparin was commenced as systemic anticoagulation to prevent circuit thrombosis, and sweep gas flow of 10 L/min was commenced to maximise carbon dioxide removal. Tidal volume reduction was performed in increments, aiming for a tidal volume≤3 mL/kg predicted body weight. The intervention was intended to be continued for at least 48 hours, and for a maximum of 7 days. Patients who were randomised to standard care were recommended to receive mechanical ventilation with a tidal volume 6 mL/kg predicted body weight, with PEEP titrated according to the ARDSNet protocol.^17^ There was no statistically significant difference between treatment arms in 90-day mortality, and the full results of the REST trial have previously been reported.^12^

### St George’s Respiratory Questionnaire (SGRQ)

To assess for an effect on long-term respiratory function, patients enrolled into the REST trial who consented to long-term follow-up were contacted to complete the SGRQ. The SGRQ is a 50-item questionnaire that has 76 weighted responses and is separated into three domains (symptoms, activity and impacts), with a lower score indicating fewer symptoms within that domain.^18^ For responders to the SGRQ, results are subdivided into the three domains. Data was included for individual domains regardless of whether the full questionnaire was completed. The SGRQ was sent by post to patients, and non-responders had follow-up contact by telephone.

### Post-traumatic stress disorder

Neuropsychiatric symptoms were evaluated using the post-traumatic stress symptoms (PTSS)-14 questionnaire. PTSS-14 is a telephone questionnaire comprised of 14 symptoms of post-traumatic stress that has been validated for use in patients who survive ICU in the UK.^19^ The PTSS-14 questionnaire was completed at 1 year after randomisation by trained study staff.

### Cognitive function

Cognitive function was assessed using the Montreal Cognitive Assessment (MoCA)-Blind scoring system. If the participant was unable to complete the MoCA-Blind questionnaire, the AD8 Score was completed by a proxy to inform the level of cognition.^20^ The MoCA-Blind questionnaire is an adapted version of the MoCA assessment tool,^21 22^ which is used to assess for cognitive dysfunction using domains that assess attention and concentration, memory, language, conceptual thinking, calculations and orientation. It is recommended as a tool to assess cognition in patients who survive ICU admission with AHRF.^23^ These questionnaires were completed by telephone at 1 year post randomisation by trained study staff.

### Health-related quality of life

Health-related quality of life was assessed in survivors at 1 year post randomisation using the EuroQol Five Dimension 5 Level (EQ-5D-5L) Questionnaire, which provides a description of health using five dimensions each with 5 levels of severity and a visual analogue scale (VAS).^24^ Responses on the descriptive system were converted into utility scores using the Crosswalk Value Set for the UK population.^25^ This tariff maps the EQ-5D-5L responses on to the EQ-5D-3L and is currently the approach recommended by the National Institute for Health and Care Excellence.^26^ To complete the EQ-5D-5L, patients were sent the questionnaires by post. Telephone completion was also used for non-responders.

If participants returned questionnaires, or answered telephone questionnaires, with concerning mental health symptoms or expressed suicidal ideation, the patient’s response was discussed with the study chief investigator, or the patient’s general practitioner if prior permission was granted for this.

### Outcomes

All outcomes reported in this manuscript were prespecified in the REST trial protocol.^18^ Mortality status was confirmed by contacting the patient’s general practitioner at 6 months, 1 year and 2 years post randomisation, prior to contacting participants for their questionnaire data. Responses to the SGRQ, PTSS-14, MoCA-Blind and EQ-5D-5L were obtained at 1 year post randomisation.

The REST trial evaluated a complex intervention that was intended to facilitate lower tidal volume mechanical ventilation. To evaluate whether there was a treatment effect based on tidal volume reduction, an exploratory analysis of patients who had a reduction in tidal volume (defined as a change from baseline to day 3 of at least 2 mL/kg PBW), compared with patients in standard care who had no reduction (defined as a change in tidal volume from baseline to day 3 no greater than 2 mL/kg PBW) was performed.

### Statistical analysis

Patients were analysed according to their randomisation group. For 6 months, 1 year and 2 years mortality risk ratios and mean difference with 95% CIs were calculated, and p values reported, from χ^2^ tests. Mortality was also analysed by survival methods with p values reported from the log-rank test and Cox proportional hazards used to estimate the HR and 95% CI. The proportionality assumption was tested using the Schoenfeld test and the assumption was satisfied. For all other outcomes and for the exploratory tidal volume analysis, the mean difference and 95% CIs were calculated and p values reported from independent samples t-tests. For the baseline characteristics median and IQR are presented and n (%) for categorical data. For all other analyses, data are presented as mean (SD) unless otherwise stated. Questionnaire responses are presented as n (%), and the denominator represents the number of patients alive at 1 year who had not withdrawn from questionnaire follow-up. In order to be able to combine the MoCA-Blind and AD8 scores, the analysis was based on level of cognitive impairment (severe, moderate, mild, normal). The MoCA-Blind was converted to a score out of 30 and then categorised as follows; <10 severe, 10–17 moderate, 18–25 mild and >25 normal cognition.^21 27^ An AD8 Score of 0–1 is considered normal cognition.^20^ In the event of missing data for the MoCA-Blind questionnaire, the total recall score was calculated based on the items answered. Level of cognition was analysed using a χ^2^ test. Additional post-hoc analyses were performed. First, to assess whether hypoxaemia and treatment allocation had an influence on the cognitive outcomes, the MoCA-Blind Score was adjusted for the lowest recorded daily PaO_2_ in the first 7 days from randomisation, and the data were analysed using analysis of covariance. Second, to assess whether there was a relationship between daily PaCO_2_ or neuromuscular blockade and health-related quality of life, correlation between these variables and EQ-5D-5L VAS Score was calculated using Pearson’s correlation coefficient. There was no imputation for missing data. Analysis was conducted using Stata/SE, V.15.1 (StataCorp). Statistical significance was defined using a two-sided test with α=0.05.

## Results

**Figure 1:**
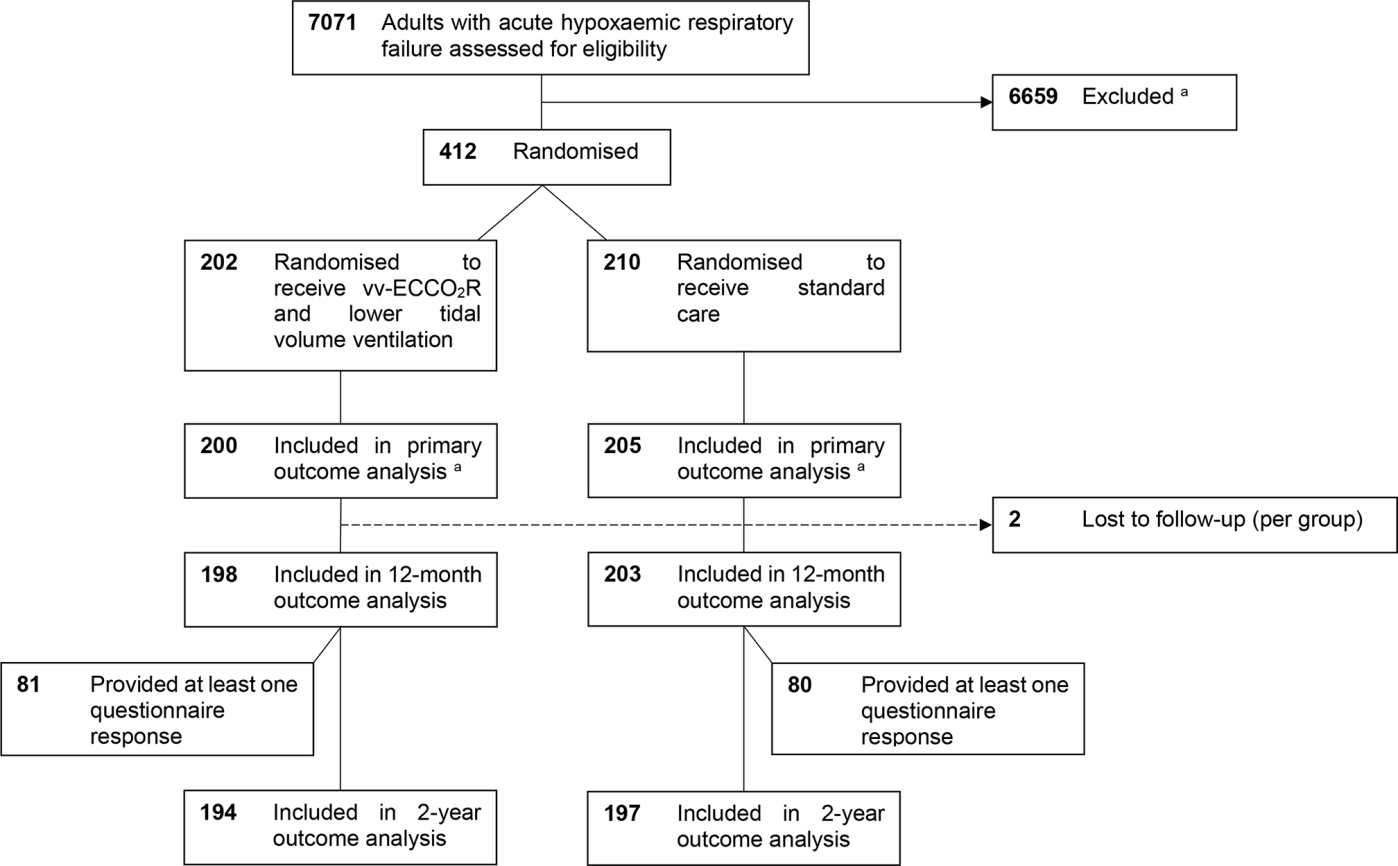
Flow of patients in the REST trial. ^a^The primary analysis, including the reasons for exclusion, has previously been reported.^12^ vv-ECCO_2_R, veno-venous extracorporeal carbon dioxide removal.

Of the 412 patients enrolled into the REST trial, 1-year mortality status was available for 401 patients (198 (49.4%) randomised to receive intervention and 203 (50.6%) randomised to receive standard care), and 2-year mortality status was available for 391 patients (194 intervention, 197 standard care). Of patients alive at 1 year, 161 provided at least one questionnaire response (figure 1). The baseline characteristics of these patients are presented in table 1. The baseline characteristics were similar to those of patients who did not complete any of the 1-year questionnaires (online supplemental table S1).

10.1136/thorax-2022-218874.supp1 Supplementary data

**Table 1:** Baseline characteristics of all patients who responded to at least one questionnaire 1 year following randomisation

	Intervention (n=81)	Standard care (n=80)	P value
Age (years)	58.5 (49.2, 67.0)	59.1 (49.4, 67.5)	0.57
Female—N (%)	31 (38%)	32 (40%)	0.82
Dependency prior to hospital admission—N (%)			0.56
Able to live without assistance	60 (86%)	62 (91%)	
Minor assistance	9 (13%)	5 (7%)	
Major assistance	1 (1%)	1 (1%)	
Total assistance	0 (0%)	0 (0%)	
Predicted body weight (kg)*	66.0 (56.9, 74.2)	66.0 (57.0, 72.4)	0.95
ICU admission diagnostic category—N (%)†			
Respiratory	67 (84%)	67 (84%)	1.00
Sepsis	30 (38%)	35 (44%)	0.42
Cardiovascular	17 (21%)	17 (21%)	1.00
Kidney	14 (18%)	15 (19%)	0.84
Gastrointestinal	13 (16%)	14 (18%)	0.83
Central nervous system	6 (8%)	7 (9%)	0.77
Other	6 (8%)	2 (3%)	0.15
Toxicology	6 (8%)	4 (5%)	0.51
Haematology	1 (1%)	1 (1%)	1.00
Orthopaedic	3 (4%)	3 (4%)	1.00
ARDS present at enrolment^†^	42/79 (53%)	44/80 (55%)	0.82
Aetiology of ARDS—N (%)‡			
Pneumonia	32 (76%)	32 (73%)	0.71
Sepsis	19 (45%)	18 (41%)	0.69
Gastric content aspiration	4 (10%)	5 (11%)	0.78
Other	4 (10%)	4 (9%)	0.95
Pancreatitis	1 (2%)	5 (11%)	0.10
Thoracic trauma	0 (0%)	1 (2%)	0.33
Smoke/toxin inhalation	2 (5%)	1 (2%)	0.53
APACHE II Score at ICU admission§	17 (14, 22)	19 (16, 22)	0.08
SOFA Score¶	9 (8, 11) n=78	10 (8, 12) n=76	0.61
Mode of ventilation—N (%)			0.20
Mandatory	68 (84%)	66 (83%)	
Mandatory and spontaneous breaths	9 (11%)	5 (6%)	
Spontaneous	4 (5%)	9 (11%)	
Adjunctive ventilatory therapies—N (%)			
Neuromuscular blocking drugs	42 (52%)	37 (46%)	0.48
Prone positioning	6 (7%)	4 (5%)	0.53
Inhaled nitric oxide	1 (1%)	0 (0%)	0.32
Nebulised epoprostenol	1 (1%)	2 (3%)	0.55
Tidal volume (mL/kg PBW)**	6.2 (5.7, 7.1)	6.3 (5.8, 7.4)	0.64
Respiratory rate (breaths/min)	24 (20, 26)	24 (20, 28)	0.38
PEEP (cm H_2_O)	10 (9, 12) n=80	10 (8, 14) n=80	0.84
Plateau pressure (cm H_2_O)	25 (22, 27) n=61	26 (24, 30) n=60	0.08
Driving pressure (cm H_2_O)††	13 (11, 17) n=60	14 (12, 18) n=60	0.09
PaO_2_/FiO_2_ ratio (mm Hg)‡‡	118.5 (96.8, 136.5) n=79	111.8 (92.3, 130.5) n=79	0.19
PaCO_2_ (mm Hg)	54.8 (47.3, 62.3) n=79	52.9 (47.2, 60.5) n=78	0.67
pH	7.33 (7.27, 7.39) n=79	7.31 (7.25, 7.38) n=78	0.16

Baseline clinical data were collected in the 24 hours prior to randomisation unless stated otherwise. If more than one value was available for this 24-hour period, the value closest but prior to the time of randomisation was recorded.

Data presented as median (IQR) unless otherwise stated. Where median (IQR) presented, p value is from a Wilcoxon rank sum. Where, N (%) presented, p value is from a χ^2^ test.

*The predicted body weight of male patients was calculated as equal to 50+0.91 (centimetres of height—152.4); that of female patients was calculated as equal to 45.5+0.91 (centimetres of height—152.4).

†Patients may have had more than one admission diagnostic category or cause of ARDS identified.

‡The presence of ARDS was assessed by the treating physician.

§Scores on the Acute Physiology and Chronic Health Evaluation (APACHE) II range from 0 to 71, with higher scores indicating greater severity of illness.

¶Scores on the Sequential Organ Failure Assessment (SOFA) scale range from 0 to 24, with higher scores indicating greater severity of disease.

**Tidal volume represents the pre-randomisation value.

††Driving Pressure = Plateau Pressure − PEEP.

‡‡Second qualifying PaO_2_/FiO_2_ ratio.

APACHE, Acute Physiology and Chronic Health Evaluation; ARDS, acute respiratory distress syndrome; FiO_2_, fraction of inspired oxygen; ICU, intensive care unit; PaCO_2_, partial pressure of arterial carbon dioxide; PaO_2_, partial pressure of arterial oxygen; PBW, Predicted Body Weight; PEEP, positive end-expiratory pressure; SOFA, Sequential Organ Failure Assessment.

After randomisation, patients allocated to intervention had a lower daily tidal volume (to day 7), and a lower PaO_2_/FiO_2_ ratio but a higher PaCO_2_ on days 2–6. Daily postrandomisation ventilatory parameters are provided in the online supplemental table S2. There was no difference in the duration of mechanical ventilation between patients allocated to intervention (17.5 (10.8) days) or standard care (21.0 (40.2); p=0.46).

### Mortality

**Figure 2:**
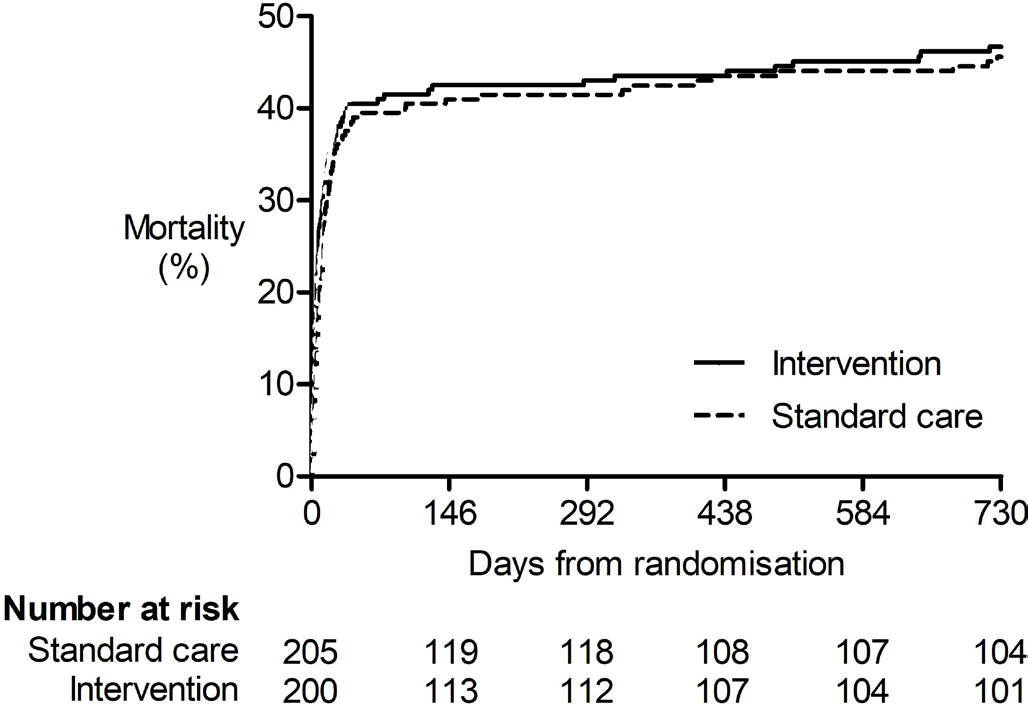
Kaplan-Meier curve of the time to death dichotomised by treatment group in patients recruited to the REST trial. There was no difference in the time to death between patients allocated to intervention or standard care (HR 1.1 (0.8, 1.4); log-rank p=0.61).

The time to death up to 2 years following randomisation was similar between patients allocated to intervention and standard care (HR 1.08 (0.81, 1.44); log-rank test p=0.61) (figure 2). Overall mortality at 6 months, 1 year and 2 years was 42.4%, 43.4% and 46.9%, respectively. There was no statistically significant difference between patients allocated to intervention or standard care in mortality at any of these timepoints (table 2).

**Table 2:** Mortality results at 6 months, 1 year and 2 years following randomisation

	Intervention, N (%)	Standard care, N (%)	% point difference (95% CI)	Risk ratio (95% CI)	P value
6-month mortality	85 (42.9%)	85 (41.9%)	1.1% (−8.6% to 10.7%)	1.0 (0.8 to 1.3)	0.83
1-year mortality	87 (43.9%)	87 (42.9%)	1.1% (−8.6% to 10.8%)	1.0 (0.8 to 1.3)	0.83
2-year mortality	93 (47.2%)	93 (47.9%)	0.7% (−9.2% to 10.6%)	1.0 (0.8 to 1.3)	0.89

Randomisation represents the start of each time period. p value derived from χ^2^ test.

### St George’s Respiratory Questionnaire

The SGRQ was completed in its entirety by 116 (53%) patients alive at 1 year who had not withdrawn from paper questionnaire follow-up. The most frequently completed domain was the SGRQ symptom score (n=129). There was no significant difference in SGRQ total score between patients allocated to intervention (40.9 (27.1)) or standard care (40.9 (26.4); p=1.00). Similarly, there was no significant difference between treatment allocation in either the symptoms (intervention 41.7 (29.8) vs standard care 45.1 (31.8)); p=0.52), activity (intervention 58.9 (31.2) vs standard care 58.2 (32.4); p=0.91) or impacts (intervention 29.7 (28.1) vs standard care 28.6 (26.2); p=0.83) component scores of the SGRQ (table 3).

**Table 3:** Questionnaires at 1 year following randomisation

	Intervention	Standard care	Mean difference (95% CI)	P value
SGRQ total score	40.9 (27.1) n=53	40.9 (26.4) n=63	−0.02 (−9.9 to 9.9)	1.00
Symptoms score	41.7 (29.8) n=62	45.1 (31.8) n=67	3.4 (−7.4 to 14.2)	0.52
Activity score	58.9 (31.2) n=57	58.2 (32.4) n=66	−0.7 (−12.1 to 10.7)	0.91
Impacts score	29.7 (28.1) n=58	28.6 (26.2) n=64	−1.1 (−10.8 to 8.7)	0.83
PTSS-14 Score	34.3 (19.8) n=60	38.8 (22.2) n=56	4.5 (−3.2 to 12.2)	0.25
MoCA-Blind Score*	17.1 (3.9) n=59	17.9 (3.1) n=56	0.8 (−0.5 to 2.1)	0.23
Normal cognition†	30 (50.0%)	27 (48.2%)		0.41
Mild cognitive impairment†	20 (33.3%)	23 (41.1%)		
Moderate cognitive impairment†	10 (16.7%)	5 (8.9%)		
Severe cognitive impairment†	0 (0.0%)	1 (1.8%)		
EQ-5D-5L utility score	0.56 (0.36) n=63	0.56 (0.34) n=67	−0.004 (−0.13 to 0.12)	0.95
EQ-5D-5L VAS	60.4 (23.6) n=66	66.8 (22.1) n=67	6.4 (−1.4 to 14.2)	0.11

Data presented as mean (SD) and analysed using independent samples t-test.

*Maximum score of 22.

†MoCA-Blind and AD8 scores were converted to level of cognition and analysed using χ^2^.

EQ-5D-5L, EuroQol Five Dimension five level; MoCA, Montreal Cognitive Assessment; PTSS, Post-traumatic stress symptoms; SGRQ, St George’s Respiratory Questionnaire; VAS, visual analogue scale.

### Post-traumatic stress disorder

Overall, 116 (56%) patients alive at 1 year who had not withdrawn from telephone questionnaire follow-up completed the PTSS-14 questionnaire, and there was a similar score between treatment groups (intervention (34.3 (19.8)) vs standard care 38.8 (22.2); p=0.25) indicating that the prevalence of post-traumatic stress disorder was similar between treatment groups (table 3).

### Cognitive function

The MoCA-Blind Questionnaire was completed by 115 (56%) patients alive at 1 year who had not withdrawn from telephone questionnaire follow-up, while for 1 patient, cognitive function was assessed via proxy using the AD-8 Questionnaire, and the results from both questionnaires were combined. At 1 year, there was no significant difference in the proportion of patients between intervention and standard care who had mild (intervention 33.3% vs standard care 41.1%), moderate (16.7% vs 8.9%) or severe (0% vs 1.8%) cognitive impairment (p=0.41) (table 3).

Likewise, rates of cognitive impairment, as measured by the MoCA-Blind questionnaire score, were similar between treatment groups (intervention 17.1 (3.9) vs standard care 17.9 (3.1); p=0.23) (table 3). Adjustment for the lowest recorded PaO_2_ did not affect these results (mean difference 0.8 (−0.2, 2.2)).

### Health-related quality of life

Health-related quality of life, assessed by both the EQ-5D-5L utility score and VAS score, was the most frequently returned questionnaire, 133 (61%) of participants alive at 1 year who had not withdrawn from paper questionnaire follow-up. Both the EQ-5D-5L utility and VAS scores were similar between patients allocated to either intervention or standard care who survived to 1 year (table 3).

There was no statistically significant correlation between daily PaCO_2_ (within the first 7 days after randomisation) and EQ-5D-5L VAS Score (online supplemental figures e1-7), nor between the number of days of neuromuscular blockade within the first 7 days after randomisation and EQ-5D-5L VAS Score (correlation coefficient −0.08; p=0.39).

### Effect of tidal volume reduction on long-term outcomes

Overall, 45 patients allocated to intervention, who completed at least one questionnaire, had a reduction in tidal volume of at least 2 mL/kg PBW between baseline and day 3. These patients were compared with 68 patients allocated to standard care who did not have at least a 2 mL/kg PBW reduction in tidal volume over the same time. As expected, at day 3, the tidal volume was lower in the intervention subgroup than the standard care subgroup (3.6 (0.7) vs 6.9 (2.0) mL/kg PBW; p<0.001).

In patients who had a reduction in tidal volume, there was no significant effect on respiratory function, cognitive dysfunction or health-related quality of life at 12 months. Although patients allocated to intervention who had a meaningful tidal volume reduction had a numerically lower PTSS-14 Score than those standard care patients with no change in tidal volume, the difference in PTSS-14 Score did not reach statistical significance (p=0.06) (table 4).

**Table 4:** Exploratory analysis evaluating the effect of tidal volume reduction

	Intervention (tidal volume reduction≥2 mL/kg PBW)	Standard care (tidal volume reduction<2 mL/kg PBW)	Mean difference (95% CI)	P value
SGRQ total score	38.9 (27.2) n=33	42.6 (26.5) n=54	3.8 (−8.0 to 15.5)	0.53
Symptoms score	41.0 (30.8) n=39	46.5 (31.0) n=58	5.5 (−7.2 to 18.2)	0.39
Activity score	56.5 (31.6) n=34	59.9 (32.5) n=57	3.4 (−10.5 to 17.2)	0.63
Impacts score	27.9 (28.5) n=37	30.2 (26.8) n=55	2.2 (−9.4 to 13.8)	0.70
PTSS-14 Score	30.4 (19.6) n=32	39.8 (22.3) n=48	9.4 (−0.2 to 19.1)	0.06
MoCA-Blind total score	17.4 (3.6) n=32	17.8 (3.1) n=48	0.4 (−1.1 to 1.9)	0.62
EQ-5D-5L utility score	0.58 (0.35) n=39	0.53 (0.34) n=58	−0.05 (−0.19 to 0.10)	0.52
EQ-5D-5L VAS	62.4 (25.1) n=40	64.5 (21.6) n=58	2.1 (−7.4 to 11.5)	0.67

Data presented as mean (SD) and analysed using independent samples t-test.

EQ-5D-5L, EuroQol Five Dimension 5 Level; MoCA, Montreal Cognitive Assessment; PBW, Predicted Body Weight; PTSS, Post-traumatic stress symptoms; SGRQ, St George’s Respiratory Questionnaire; VAS, visual analogue scale.

## Discussion

Understanding the effect of an intervention on long-term mortality and the long-term physical, neurological and psychological impact on patients is an important component to interpreting the results of a randomised controlled trial. In the REST trial, although the intervention of vv-ECCO_2_R to facilitate lower tidal volume ventilation did not reduce 90-day mortality,^12^ it was feasible that there could have been benefits to patients who were only identified at a later time period. However, in this prespecified analysis,^16^ it has been demonstrated that the intervention studied in the REST trial did not reduce mortality at either 6 months or 1 year from randomisation. Furthermore, in the patients who provided questionnaire responses, lower tidal volume ventilation, facilitated by vv-ECCO_2_R, did not significantly reduce the long-term physical or neuropsychological symptom burden, nor improve their health-related quality of life. These data do not support the use of vv-ECCO_2_R to facilitate lower tidal volume ventilation in patients with moderate-to-severe AHRF.

In the REST trial, patients allocated to intervention had a 90-day mortality rate of 41.5%, while in patients allocated to standard care 90-day mortality was 39.5%. There was a small increase in mortality between 90 days and 1 year in these patients (2.4% increase in patients allocated to intervention, 3.4% increase in patients allocated to standard care), and the time to death was similar between groups. The small increase in mortality over time was lower than has been previously reported. Most studies of patients with ARDS demonstrate an increase in mortality over the first year of between 8% and 17%.^28–30^ Furthermore, pneumonia and sepsis were the most common reasons for ICU admission in this study, and a previous study of patients with sepsis demonstrated a 6%–8% increase in the annual mortality rate in patients who survived critical care admission.^31^ The REST trial recruited patients with moderate-to-severe AHRF and it is possible that the small increase in late mortality over the first year in this study is related to a higher 90-day mortality in a population with greater disease severity at inclusion.

In an effort to better understand the role that tidal volume reduction had on long-term outcomes, patients allocated to intervention who had a tidal volume reduction of at least 2 mL/kg PBW were compared with those patients in standard care who did not have this change in tidal volume. The results from this subgroup analysis did not demonstrate a statistically significant difference in self-reported respiratory function, neuropsychological symptom burden or health-related quality of life. However, patients allocated to intervention who had a tidal volume reduction of at least 2 mL/kg PBW had a numerically lower PTSS-14 than patients allocated to standard care who had no change in tidal volume. Although this difference did not reach statistical significance, it remains a potentially important difference between these groups that may warrant further study. Previously, it has been demonstrated that lower tidal volume ventilation is associated with less hippocampal apoptosis in a preclinical model.^10^ However, in this subgroup analysis, there was no difference in cognitive dysfunction (as assessed using the MoCA-Blind questionnaire) at 1 year between treatment groups, suggesting that this mechanism of injury may not be clinically significant in this patient cohort.

In comparison to previously described cohorts of patients with ARDS, the SGRQ scores were numerically higher (indicating a higher symptom burden) in survivors at 1 year in the REST trial when compared with previously described cohorts of patients with ARDS.^32–34^ Interestingly, the recorded values in the REST trial are also higher than those described for patients with COPD and asthma.^35^ Further evaluation of the long-term physical consequences of AHRF is warranted to improve understanding and identify therapies that can modulate long-term outcomes in these patients.

This planned analysis of the REST trial has several strengths. The extended evaluation of mortality to 1 year provides important data that excludes a later treatment effect, which may have been missed had follow-up ceased at 90 days. Patients with AHRF are recognised as having an increased risk of mortality beyond their hospital discharge,^2^ and therefore longer-term follow-up of these patients allows investigators to identify a late trend in outcomes which may influence the overall interpretation of the clinical trial results. In addition, the 1-year mortality rate in the REST trial is comparable to that observed in patients with ARDS,^3 29 36^ suggesting that the long-term outcomes presented in this manuscript are in keeping with that of a broader population of patients. The 1-year symptom and functional assessments were conducted using validated questionnaires many of which are recommended in the core outcome set for this population.^23^ Evaluating this broad set of outcomes supports the mortality results and provides an opportunity to identify differences in morbidity and quality of life that may affect survivors and therefore influence the interpretation of results from a clinical trial. Finally, because the REST trial evaluated a complex intervention that was intended to facilitate lower tidal volume ventilation, there were patients who were allocated to intervention but did not have the intended reduction in tidal volume. In performing a subgroup analysis which evaluated patients with a reduction in tidal volume of at least 2 mL/kg PBW, we sought to establish whether intervention fidelity was associated with outcomes. The absence of an observed benefit in this cohort strengthens the overall findings of this study, highlighting that the absence of an effect was unlikely to be a consequence of low intervention fidelity.

Despite these aspects there are some limitations to consider. First, only 40% of eligible patients provided responses to the questionnaires, while the most frequently completed questionnaire was informed by 33% of the eligible population. The incomplete response rate to questionnaires is likely to be multifactorial and is consistent with previous studies of ICU survivors.^28 37^ This is a significant limitation that means the results are subject to response bias, and therefore they may not fully reflect the health status of all survivors to 1 year. Second, although validated for their use, the questionnaires were self-reported, and are therefore subject to bias, including that the postal questionnaires were completed by a proxy rather than the patient themselves. To overcome this, an objective assessment of physical function, such as the 6 min walk test, could have been considered. Although the core outcome set for studies evaluating survivors of acute respiratory failure was published after the REST trial had commenced,^23^ it remains a weakness that there are some domains from this recommendation that were not covered in this long-term analysis. In addition, data regarding a patient’s capacity to return to work would have been useful; however, these data were not available. This highlights the need to collect multidimensional long-term outcomes in future trials. Finally, the intervention was designed to use vv-ECCO_2_R to facilitate lower tidal volume ventilation, with the aim of reducing ventilator-induced lung injury. The ECCO_2_R device used in the trial has a centrifugal pump.^38^ It remains uncertain whether devices using differing technology with higher flow rates and with higher CO_2_ removal capacity which could facilitate further reductions in injurious ventilation than achieved in the REST trial, would be associated with clinical benefit. In the absence of a biological difference between the groups, it is implausible that there will be a difference in clinical outcomes. Identification of whether there was a difference in the biological response between patients allocated to intervention and standard care is a potentially important step to understanding why there was no difference in short-term and long-term outcomes between intervention and standard care.

In summary, these data demonstrate that lower tidal volume ventilation, facilitated by vv-ECCO_2_R as delivered in the REST trial, does not reduce long-term mortality in patients with moderate-to-severe AHRF, when compared with standard care. These data reinforce that vv-ECCO_2_R with lower tidal volume ventilation strategy should not be used routinely in this patient cohort. Further clinical trials are required to determine if devices with higher CO_2_ removal capacity to facilitate further reductions in injurious ventilation are associated with clinical benefit.

## Data Availability

Data are available upon reasonable request.
