# Supplementary material for: Acute hypoxaemic respiratory failure after treatment with lower tidal volume ventilation facilitated by extracorporeal carbon dioxide removal: long-term outcomes from the REST randomised trial
Source: Thorax. 2022 Oct 5;78(8):767–74. doi: 10.1136/thorax-2022-218874 (PMC10359587; doi:10.1136/thorax-2022-218874)
Supplement: Supplementary data [file thorax-2022-218874supp001.pdf]

Acute hypoxaemic respiratory failure after treatment with lower tidal volume ventilation facilitated by extracorporeal carbon dioxide removal: long-term outcomes from the REST randomised trial

Andrew J Boyle [1,2], Clíona McDowell [3], Ashley Agus [3], Danielle Logan [3], Jonathan D Stewart [1], Colette Jackson [3], Jeanette Mills [3] James J McNamee [2], Daniel F McAuley [1,2]

Supplementary appendix

Table S1: Baseline characteristics of patients that did not respond to any questionnaires 1-year following randomisation

	**Intervention** (n = 121)	**Standard care** (n = 130)
**Age** (years)	61.6 [51.6, 71.7]	63.5 [50.8, 69.9]
**Female** – N (%)	33 (27%)	47 (36%)
**Dependency prior to hospital admission** – N (%)		
Able to live without assistance	92 (88%)	98 (86%)
Minor assistance	10 (10%)	14 (12%)
Major assistance	1 (1%)	2 (2%)
Total assistance	1 (1%)	0 (0%)
**Predicted body weight** (kg) [b]	66.9 [59.7, 73.3]	66.0 [55.2, 71.5]
**ICU admission diagnostic category** – N (%) [c]		
Respiratory	108 (90%)	111 (85%)
Sepsis	56 (47%)	67 (52%)
Cardiovascular	28 (23%)	32 (25%)
Kidney	25 (21%)	27 (21%)
Gastrointestinal	14 (12%)	17 (13%)
Central Nervous System	11 (9%)	7 (5%)
Other	8 (7%)	8 (6%)
Toxicology	4 (3%)	6 (5%)
Haematology	6 (5%)	9 (7%)
Orthopaedic	1 (1%)	5 (4%)
**ARDS present at enrolment** [d]	76/120 (63%)	86/127 (68%)
**Aetiology of ARDS** – N (%) [c]		
Pneumonia	64 (84%)	71 (83%)
Sepsis	35 (46%)	48 (56%)
Gastric content aspiration	4 (5%)	9 (10%)
Other	4 (5%)	6 (7%)
Pancreatitis	4 (5%)	0 (0%)
Thoracic trauma	2 (3%)	2 (2%)
Smoke / toxin inhalation	0 (0%)	1 (1%)
**APACHE II score at ICU Admission** [e]	19 [15,24]	21 [16,24]
**SOFA score** [f]	10 [7,13] n=117	10 [7,12] n=122

**Mode of Ventilation** – N (%)		
Mandatory	90 (75%)	97 (75%)
Mandatory and spontaneous breaths	28 (23%)	26 (20%)
Spontaneous	2 (2%)	6 (5%)
**Adjunctive ventilatory therapies** – N (%)		
Neuro-Muscular Blocking Drugs	61 (51%)	65 (50%)
Prone Positioning	16 (13%)	19 (15%)
Inhaled Nitric Oxide	5 (4%)	4 (3%)
Nebulized Epoprostenol	2 (2%)	3 (2%)
**Tidal volume** (mL/kg PBW)	6.3 [5.9,7.0] n=120	6.5 [5.9,7.0] n=128
**Respiratory rate** (breaths/min)	24 [20,28] n=120	24 [20,26] n=129
**PEEP** (cm H_2O)	10 [8,12] n=120	10 [8,12] n=128
**Plateau pressure** (cm H_2O)	27 [24,30] n=99	27 [23,30] n=103
**Driving pressure** (cm H_2O) [g]	15 [12,20] n=99	16 [13,21] n=103
**PaO_2 / FiO_2 ratio** (mmHg) [h]	117.8 [93.8,134.3] n=119	116.3 [97.9,133.5] n=124
**$PaCO_2$** (mmHg)	53.3 [47.3,64.5] n=119	55.2 [48.8,62.7] n=125
**pH**	7.30 [7.23,7.36] n=119	7.30 [7.24,7.37] n=124

Data presented as median [interquartile range] unless otherwise stated.

Abbreviations: ARDS, Acute Respiratory Distress Syndrome; APACHE, Acute Physiology and Chronic Health Evaluation; SOFA, Sequential Organ Failure Assessment; PEEP, positive end-expiratory pressure; FiO_2, fraction of inspired oxygen; PaO_2, partial pressure of arterial oxygen; $PaCO_2$, partial pressure of arterial carbon dioxide.

a. Baseline clinical data were collected in the 24 hours prior to randomisation unless stated otherwise. If more than one value was available for this 24-hour period, the value closest but prior to the time of randomisation was recorded.

b. The predicted body weight of male patients was calculated as equal to 50+0.91 (centimetres of height-152.4); that of female patients was calculated as equal to 45.5+0.91 (centimetres of height-152.4)

c. Patients may have had more than one admission diagnostic category or cause of ARDS identified.

d. The presence of ARDS was assessed by the treating physician.

e. Scores on the Acute Physiology and Chronic Health Evaluation (APACHE) II range from 0 to 71, with higher scores indicating greater severity of illness.

f. Scores on the Sequential Organ Failure Assessment (SOFA) scale range from 0 to 24, with higher scores indicating greater severity of disease.

g. Driving Pressure = Plateau Pressure - PEEP

h. Second qualifying PaO2/FiO2 ratio.

Table S2: Daily ventilation parameters

	Intervention	Standard care	Mean Difference (95% CI)
**Tidal volume mL/kg PBW**			
Day 2	4.5(1.8) n= 81	6.5(1.8) n= 79	2.0(1.4, 2.6)
Day 3	4.4(2.0) n= 79	6.7(2.0) n= 76	2.3(1.7, 3.0)
Day 4	5.2(2.5) n= 79	7.0(1.8) n= 72	1.8(1.1, 2.5)
Day 5	5.7(2.4) n= 75	7.0(2.1) n= 70	1.3(0.6, 2.0)
Day 6	6.0(2.4) n= 70	7.1(2.1) n= 69	1.2(0.4, 1.9)
Day 7	6.3(2.6) n= 66	7.1(1.8) n= 65	0.8(0.0 1.6)
**PaO_2/FiO_2 ratio (mmHg)**			
Day 1	151.5(52.3) n= 81	144.3(41.6) n= 80	-7.2(-22.0, 7.5)
Day 2	151.2(48.2) n=81	172.2(68.6) n= 79	21.1(2.6, 39.6)
Day 3	157.3(47.4) n= 79	177.7(66.8) n= 77	20.5(2.2, 38.7)
Day 4	166.4(58.5) n=79	182.9(64.9) n= 74	16.6(-3.2, 36.3)
Day 5	172.3(58.3) n= 75	186.8(60.8) n= 72	14.5(-4.9, 34.0)
Day 6	184.8(72.3) n= 73	201.4(72.3) n= 71	16.6(-7.2, 40.5)
Day 7	204.4(76.7) n= 69	201.0(68.3) n= 70	-3.3(-27.7, 21.0)
**PEEP (cm H_2O)**			
Day 1	11.3 (2.8) n= 81	11.0 (3.0) n= 80	-0.3(-1.2, 0.6)
Day 2	11.4 (3.0) n= 80	11.1 (2.8) n= 78	-0.2(-1.2, 0.7)
Day 3	11.5(3.2) n= 78	10.1 (2.9) n= 76	-1.3(-2.3, -0.4)
Day 4	11.1(3.4) n= 77	9.8(2.8) n= 71	-1.3(-2.3, -0.2)
Day 5	10.4(3.5) n=71	9.4(2.5) n= 71	-0.9(-1.9, 0.1)
Day 6	9.6(3.3) n= 68	9.3(2.6) n= 69	-0.3(-1.3, 0.7)
Day 7	9.5(3.6) n= 62	9.0(2.5) n= 65	-0.5(-1.6, 0.6)
**Plateau Pressure (cm H_2O)**			
Day 1	24.7(4.8) n= 61	26.0(5.3) n= 57	1.4(-0.5, 3.2)
Day 2	22.9(5.2) n= 59	25.4(5.0) n= 59	2.6(0.7, 4.4)
Day 3	22.6(5.4) n=59	22.8(5.6) n= 58	0.1(-1.9, 2.2)
Day 4	22.2(5.9) n= 59	22.4(6.0) n= 44	0.3(-2.1, 2.6)
Day 5	22.3(6.0) n= 49	23.2(6.7) n= 40	0.9(-1.8, 3.6)
Day 6	21.7(5.6) n= 46	22.0(6.5) n=35	0.3(-2.4, 3.0)
Day 7	19.9(6.0) n= 38	21.6(6.5) n= 34	1.7(-1.3, 4.6)
**Driving Pressure (cm H_2O)**			

	Intervention	Standard care	Mean Difference (95% CI)
Day 1	13.5(5.3) n= 61	15.2(4.9) n= 57	1.7 (-0.1, 3.6)
Day 2	11.5(4.7) n= 58	14.3(5.5) n =58	2.8 (0.9, 4.6)
Day 3	11.1(4.8) n= 59	12.7(4.9) n= 57	1.6 (-0.2, 3.4)
Day 4	10.8(5.5) n= 58	12.6(5.1) n= 43	1.8 (-0.4, 3.9)
Day 5	12.0 (6.0) n= 48	13.8 (5.6) n= 40	1.9 (-0.6, 4.4)
Day 6	12.2(5.6) n= 46	12.3(6.1) n= 35	0.1 (-2.5, 2.7)
Day 7	10.6 (6.0) n= 38	12.2 (5.2) n= 34	1.6 (-1.1, 4.2)
**$PaCO_2$ (mmHg)**			
Day 1	54.3(12.3) n= 80	55.6(11.8) n= 80	1.2(-2.5, 5.0)
Day 2	61.5(13.7) n= 81	53.1(11.2) n= 79	-8.4(-12.3, -4.5)
Day 3	61.2(14.7) n= 79	52.7(11.5) n= 77	-8.5(-12.7, -4.3)
Day 4	58.4(1.9) n= 79	51.7(11.8) n= 75	-6.7(-11.3, -2.1)
Day 5	55.5(17.7) n= 76	49.8(11.9) n= 73	-5.7(-10.6, -0.7)
Day 6	52.3(13.9) n= 74	47.9(11.6) n= 72	-4.3(-8.5, -0.1)
Day 7	50.2(14.0) n= 71	47.1(11.6) n= 71	-3.1(-7.4, 1.1)
**Respiratory Rate**			
Day 1	24.0(4.7) n= 81	24.1(5.6) n= 80	0.05 (-1.6, 1.7)
Day 2	26.5(5.7) n= 81	24.8(6.2) n= 79	-1.7(-3.5, 0.2)
Day 3	27.4(6.3) n= 80	24.6(6.5) n=77	-2.8(-4.8, -0.8)
Day 4	26.5(6.8) n= 80	24.7(7.0) n= 75	-1.7(-3.9, 0.5)
Day 5	25.3(7.4) n= 78	24.6(6.6) n= 74	-0.7(-2.9, 1.6)
Day 6	25.0(8.1) n= 75	24.8(7.4) n= 73	-0.3(-.28, 2.3)
Day 7	25.8 (8.0) n= 73	24.5 (7.7) n= 70	-1.3(-3.9, 1.3)
**pH**			
Day 1	7.3(0.09) n= 79	7.3(0.11) n= 80	-0.02(-0.05, 0.01)
Day 2	7.3(0.08) n= 76	7.3(0.08) n= 77	0.05(0.02, 0.07)
Day 3	7.3(0.07) n= 74	7.4(0.08) n= 75	0.04(0.01, 0.06)
Day 4	7.4(0.09) n= 79	7.4(0.09) n= 75	0.02(-0.01, 0.04)
Day 5	7.4(0.1) n=76	7.4(0.08) n= 73	0.01(-0.02, 0.04)
Day 6	7.4(0.09) n= 74	7.4(0.08) n= 72	0.01(-0.01, 0.04)
Day 7	7.4(0.08) n= 71	7.4(0.07) n= 71	0.01(-0.01, 0.04)
**Minute Volume**			
Day 1	9.2(2.6) n=81	9.8(2.3) n=80	0.6(-0.2, 1.4)

	Intervention	Standard care	Mean Difference (95% CI)
Day 2	7.4(2.2) n=81	10.1(2.5) n= 79	2.7(2.0, 3.4)
Day 3	7.4(2.5) n=79	10.2(3.0) n= 76	2.8(1.9, 3.7)
Day 4	8.3(3.1) n= 79	10.7(3.1) n= 72	2.4(1.4, 3.4)
Day 5	8.6 (3.1) n= 75	10.9(3.6) n= 70	2.3(1.2, 3.4)
Day 6	8.8(3.0) n= 70	11.3(4.2) n= 69	2.5(1.3, 3.7)
Day 7	9.6(3.2) n= 66	10.8(3.3) n= 65	1.2(0.1, 2.3)
**CO_2 removal (ml/minute)**			
Day 1	51.1(41.7) n=64		
Day 2	82.8(37.2) n= 74		
Day 3	86.8(36.5) n= 73		
Day 4	74.9(40.9) n= 68		
Day 5	66.2(44.4) n= 63		
Day 6	54.2(46.2) n= 53		
Day 7	44.1(44.1) n= 48		

Data presented as mean (SD) and analysed using independent samples t-test.

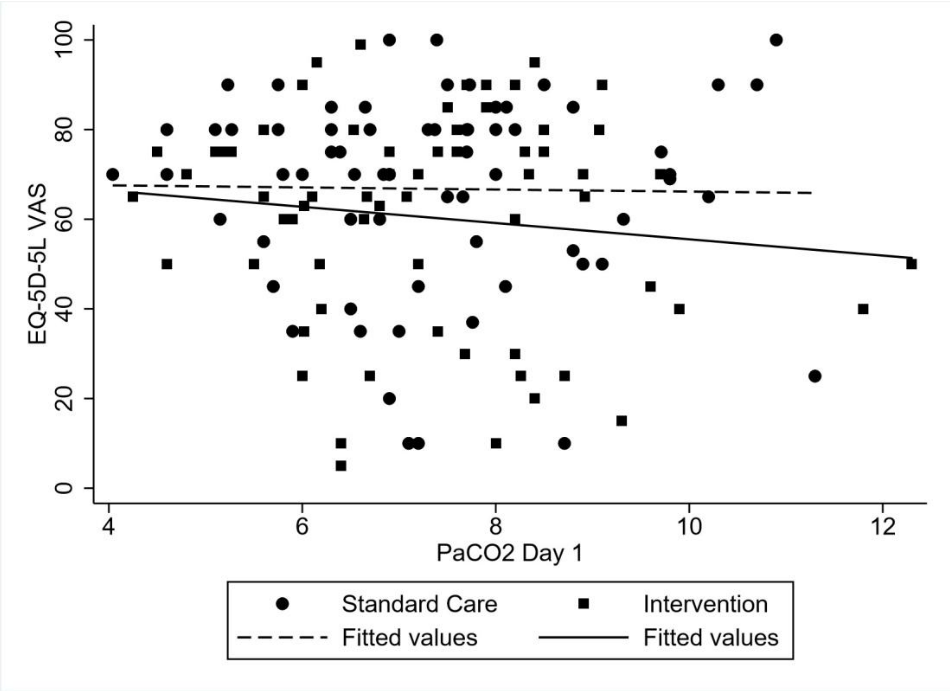

eFigure 1: Correlation between day 1 $PaCO_2$ and EQ-5D-5L VAS score. There was no correlation between day 1 $PaCO_2$ EQ-5D-5L VAS score (pearson R -0.08; p =0.38). Data analysed using Pearson's correlation coefficient.

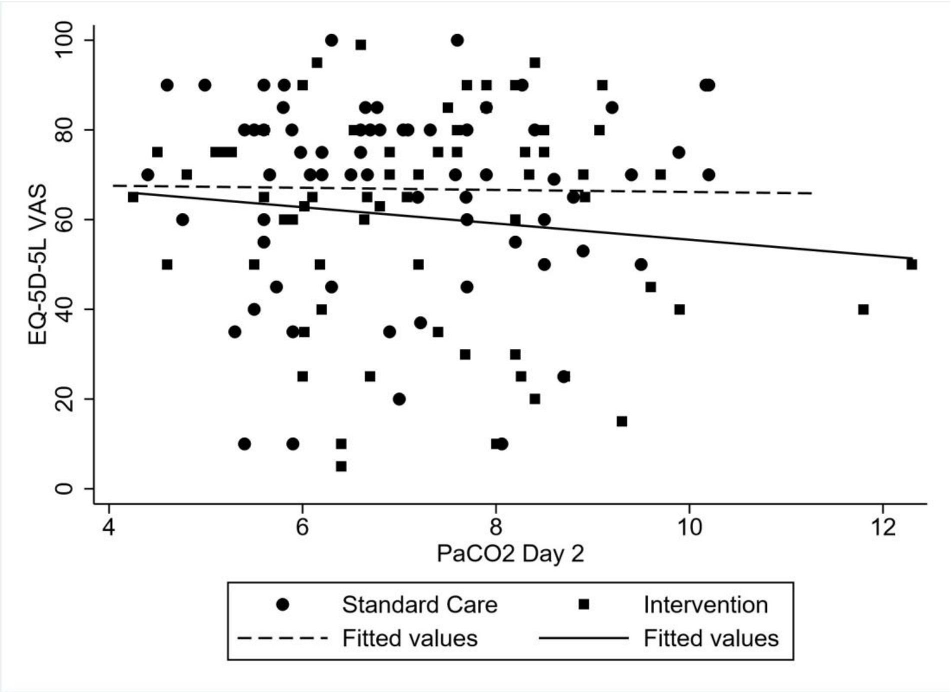

eFigure 2: Correlation between day 2 $PaCO_2$ and EQ-5D-5L VAS score. There was no correlation between day 2 $PaCO_2$ EQ-5D-5L VAS score (pearson R -0.02; p =0.86). Data analysed using Pearson's correlation coefficient.

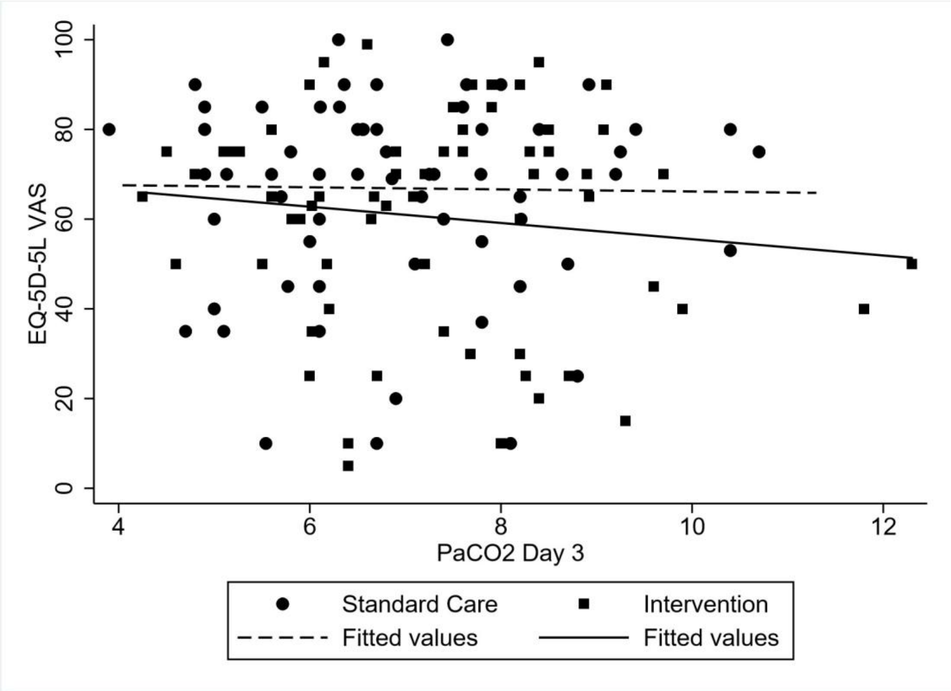

eFigure 3: Correlation between day 3 $PaCO_2$ and EQ-5D-5L VAS score. There was no correlation between day 3 $PaCO_2$ EQ-5D-5L VAS score (pearson R -0.05; p =0.57). Data analysed using Pearson's correlation coefficient.

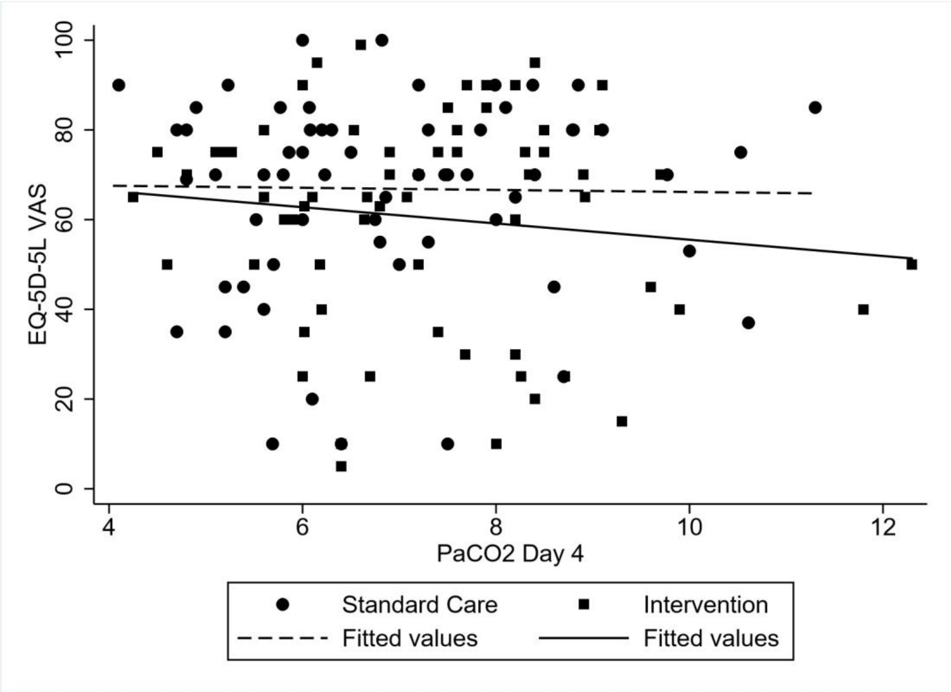

eFigure 4: Correlation between day 4 $PaCO_2$ and EQ-5D-5L VAS score. There was no correlation between day 4 $PaCO_2$ EQ-5D-5L VAS score (pearson R 0.06; p =0.53). Data analysed using Pearson's correlation coefficient.

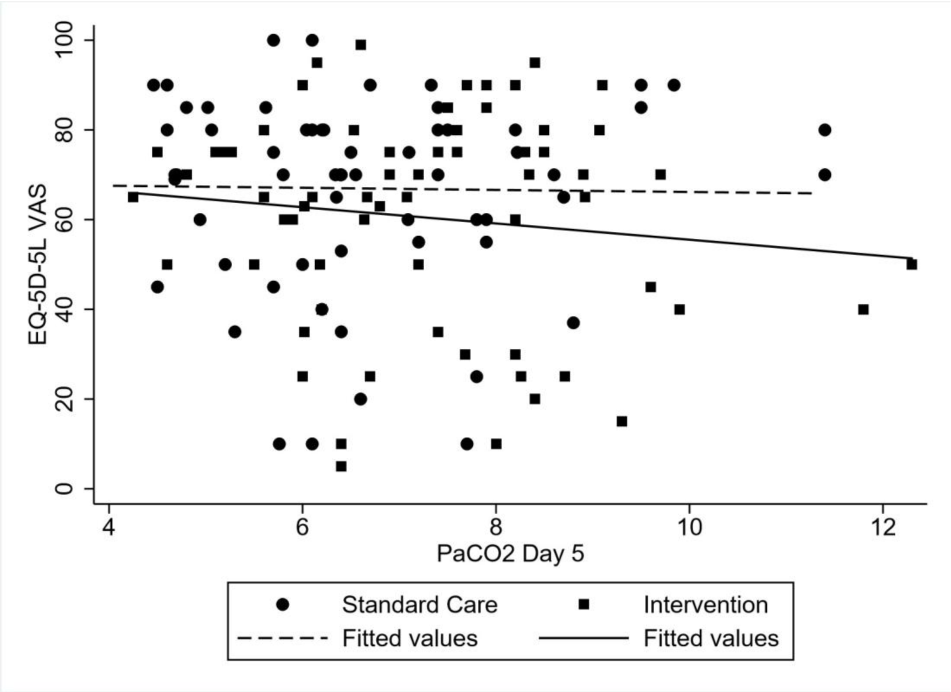

eFigure 5: Correlation between day 5 $PaCO_2$ and EQ-5D-5L VAS score. There was no correlation between day 5 $PaCO_2$ EQ-5D-5L VAS score (pearson R -0.01; p =0.88). Data analysed using Pearson's correlation coefficient.

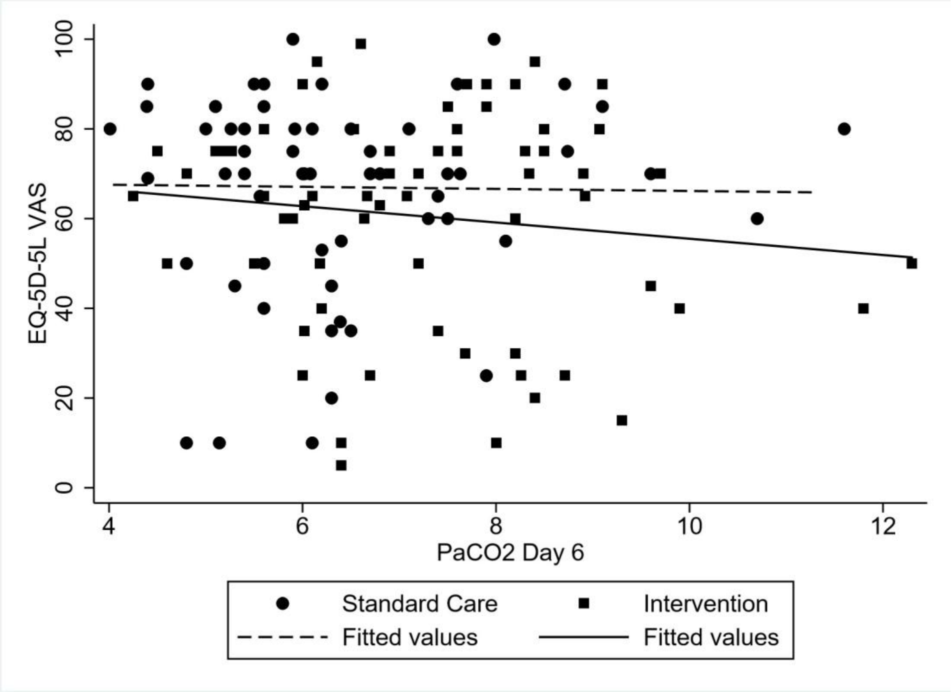

eFigure 6: Correlation between day 6 $PaCO_2$ and EQ-5D-5L VAS score. There was no correlation between day 6 $PaCO_2$ EQ-5D-5L VAS score (pearson R -0.02; p =0.80). Data analysed using Pearson's correlation coefficient.

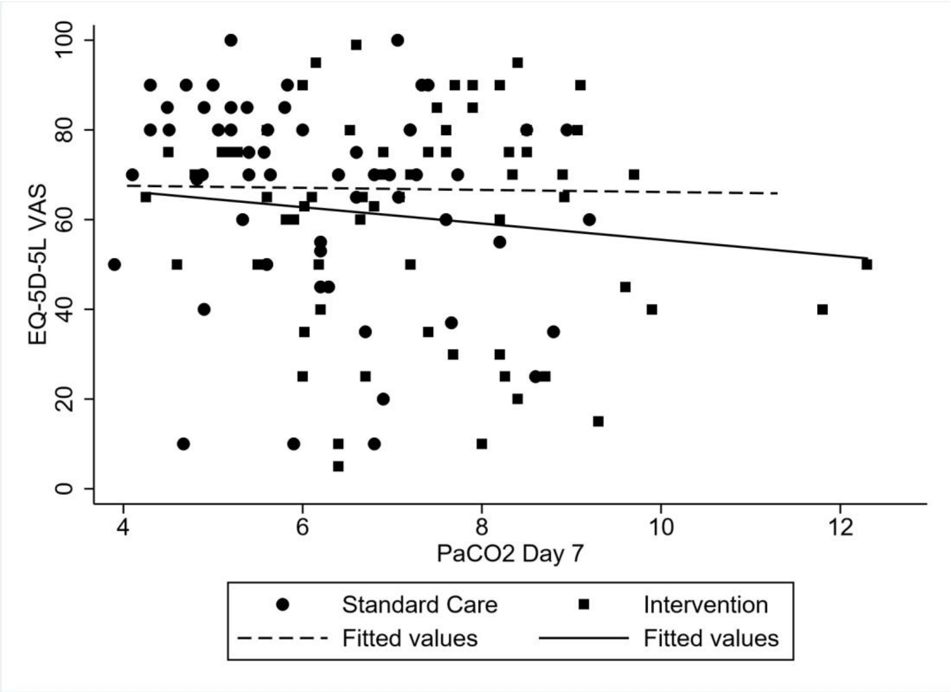

eFigure 7: Correlation between day 7 $PaCO_2$ and EQ-5D-5L VAS score. There was no correlation between day 7 $PaCO_2$ EQ-5D-5L VAS score (pearson R -0.18; p =0.06). Data analysed using Pearson's correlation coefficient.
